# Chemometric Approach Based on Explainable AI for Rapid Assessment of Macronutrients in Different Organic Fertilizers Using Fusion Spectra

**DOI:** 10.3390/molecules28020799

**Published:** 2023-01-13

**Authors:** Mahamed Lamine Guindo, Muhammad Hilal Kabir, Rongqin Chen, Jing Huang, Fei Liu, Xiaolong Li, Hui Fang

**Affiliations:** 1 College of Biosystems Engineering and Food Science, Zhejiang University, 866 Yuhangtang Road, Hangzhou 310058, China; 2 Key Laboratory of Spectroscopy Sensing, Ministry of Agriculture and Rural Affairs, Hangzhou 310058, China; 3 Huzhou Institute of Zhejiang University, 819 Xisaishan Road, Huzhou 313000, China

**Keywords:** chemometrics, phosphorous, potassium, data fusion, explainable AI, spectroscopy

## Abstract

Wet chemical methods are usually employed in the analysis of macronutrients such as Potassium (K) and Phosphorus (P) and followed by traditional sensor techniques, including inductively coupled plasma optical emission spectrometry (ICP OES), flame atomic absorption spectrometry (FAAS), graphite furnace atomic absorption spectrometry (GF AAS), and inductively coupled plasma mass spectrometry (ICP-MS). Although these procedures have been established for many years, they are costly, time-consuming, and challenging to follow. This study studied the combination of laser-induced breakdown spectroscopy (LIBS) and visible and near-infrared spectroscopy (Vis-NIR) for the quick detection of PK in different varieties of organic fertilizers. Explainable AI (XAI) through Shapley additive explanation values computation (Shap values) was used to extract the valuable features of both sensors. The characteristic variables from different spectroscopic devices were combined to form the spectra fusion. Then, PK was determined using Support Vector Regression (SVR), Partial Least Squares Regression (PLSR), and Extremely Randomized Trees (Extratrees) models. The computation of the coefficient of determination (R^2^), root mean squared error (RMSE), and residual prediction deviation (RPD) showed that FUSION was more efficient in detecting P (R^2^p = 0.9946, RMSEp = 0.0649% and RPD = 13.26) and K (R^2^p = 0.9976, RMSEp = 0.0508% and RPD = 20.28) than single-sensor detection. The outcomes indicated that the features extracted by XAI and the data fusion of LIBS and Vis-NIR could improve the prediction of PK in different varieties of organic fertilizers.

## 1. Introduction

The excessive use of chemical fertilizers in the agricultural sector has led to nutrient pollution. This pollution is one of the world’s most tenacious, costly, and complex environmental issues [1,2]. In order to rectify the situation, researchers have thought of adopting organic fertilizers, which are more environmentally friendly. Organic fertilizers can improve soil texture, water retention, and erosion reduction. The nutrients in organic fertilizers are available in a form the plant can use, aiding plant growth while causing no root burn or soil microorganism destruction. Compared to chemical fertilizer, which is more industrialized and tightly regulated, organic fertilizers are typically made domestically utilizing various basic materials. These types of fertilizers pose a problem since the production in a domestic way does not allow strict control of the different nutrients, particularly phosphorus (P) and potassium (K). These two macronutrients are essential in fertilizers because they promote plant growth but can also be a source of problems based on their amount. Indeed, a high PK content might cause nutrient pollution, especially water (surface or ground) contamination [3], while a low level can cause a reduction in product quality [4]. It is important to note that P is not only an essential plant macronutrient but also a nonrenewable and strategic resource [5]. Agricultural production depends heavily on phosphate rock, which has been the principal source of phosphorus for the past 50 years. However, phosphate rock reserves are limited. Therefore, a shortage of P may pose a threat to food production and security [5].

Given the above main problems, a rapid technique to characterize their amount is crucial for sustainable PK management.

In place of a long chemical process combined with traditional sensors techniques such as FAAS, GF-AAS, ICP-OES, and ICP-MS, sensor techniques such as laser-induced breakdown spectroscopy (LIBS) and visible and near-infrared reflectance spectroscopy (Vis-NIR) enable the rapid screening of the PK content in various organic fertilizers. LIBS is an emission spectroscopy technique where atoms and ions are created in exciting conditions due to the interaction between a tightly focused laser beam and the material sample. This technology has become popular as it can analyze different materials, such as food, soil, and fertilizer [6,7,8]. Conversely, Vis-NIR is another alternative tool that offers more and better advantages than standard chemistry. This method is non-invasive, has a high penetration rate of radiation beams, is suitable for inline use, and does not require sample preparation [9]. It has also been used to analyze numerous samples, such as food and soil [10,11]. Both methods are inexpensive, easy, non-destructive, and rapid to execute. However, they generate a tremendous amount of data, necessitating the application of chemometrics.

Chemometrics involves utilizing mathematical techniques to extract useful information and knowledge from chemical data. In previous works, the prediction of P in fertilizer with the LIBS approach primarily depended on simple chemometrics techniques such as the calibration curve method, which employed simple linear regression. This technique is unfit for the quantitative analysis of complicated samples because of the matrix effect, leading to the development of alternative chemometric techniques such as the partial least squares method for regression (PLSR), a robust technique for performing quantitative spectral analysis by selecting latent variables, and the support vector machine (SVM), a method based on hyperplanes separation. Using LIBS and PLSR, Zhang et al. [12] suggested a univariate and multivariate analysis of P elements in fertilizers. Their findings suggested that the LIBS methodology paired with PLSR may be a dependable and accurate tool for quantitatively measuring P elements in complicated samples compared to the calibration curve. Nicolodelli et al. [13] proposed a single-pulse (SP) and a double-pulse (DP) LIBS to detect P and K at different emission lines (PI = 214.91, 213.61, 255.3 nm, and KI = 769.9 nm) using PLSR. An improved support vector machine for regression was combined with LIBS to detect NPK at different ranges of 740–890 nm for N, 210–260 nm, 770–885 nm for P, and 400–410 nm, 770–885 nm for K [14]. The authors compared four different optimization techniques: the grid search method (GSM), genetic algorithm (GA), particle swarm optimization (PSO), and least squares (LS). They discovered that LS-SVM was the most accurate. Vis-NIR technology was used by Lin et al. [15] to detect P and K in chemical fertilizers. The authors first applied a genetic algorithm to extract the relevant features and used PCR for modeling. In addition, Vis-NIR has been combined with a competitive adaptive reweighted sampling algorithm (CARS) and extreme learning machine (ELM) to detect PK. [16]. However, it can be noticed that the previous works focused on chemical fertilizer; organic fertilizer has received no or little attention. In addition, LIBS has limited utility and attractiveness due to its significantly lower measurement precision. Vis-NIR may be limited under specific situations, such as extremely high or low concentrations. Consequently, further optimization strategies are required to enhance the efficacy of these two technologies.

The spectral data fusion approach is an innovative technology that merges many spectral signals [17,18]. By providing complementary information, the combined spectral data can boost the accuracy of the prediction. There are many different types of data fusion, and the specific techniques used can vary depending on the application and the data being fused. So far, spectral fusion has shown great promise in a wide range of applications, including the identification of soil components [19,20], the identification of hybrid rice [21], and the evaluation of aromatic plants [22]. To our knowledge, there have been no prior attempts to combine LIBS and Vis-NIR for PK assessment in different varieties of organic fertilizers. Moreover, there is no work using explainable AI (XAI) to extract LIBS and Vis-NIR essential features. The concept of XAI is developing faster because it is no longer just a question of predicting and having the result, but the model must be understood and interpreted by humans. This technique has been used in medical fields such as drug discovery [23] and diagnostics [24] and other fields such as finance [25] and industry [26].

Consequently, this study aimed to examine the effectiveness of Vis-NIR, LIBS, and spectral fusion to predict PK in different organic fertilizers using XAI. The specific objectives were: (i) to compare LIBS and Vis-NIR to determine PK in different organic fertilizers; (ii) to use XAI to explain the best model and extract features for LIBS and Vis-NIR. (iii) to propose a spectral data fusion and compare the result of single- and multi-sensor detection.

## 2. Results and Discussion

### 2.1. Descriptive Analysis

**Figure 1:**
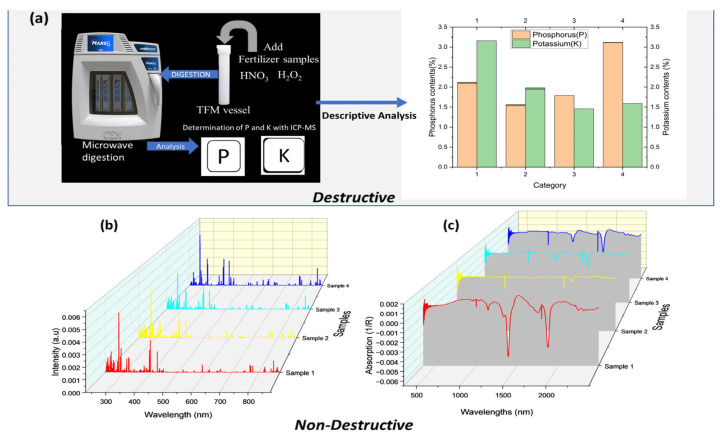
Organic fertilizer data; (**a**) Actual values obtained by ICP-MS; (**b**) preprocessed data obtained by LIBS; (**c**) Preprocessed data obtained by Vis-NIR.

The different concentrations of P and K determined with ICP-MS for the diverse variety are shown in Figure 1a. The descriptive statistics demonstrated that P’s highest concentration was category four, and the lowest was two. The central tendency (Mean) measure was 1.53%, while the standard deviation (Std) was 0.58%. The lower quartile (Q1) and the upper quartile(Q3) were estimated to be 1.71% and 2.34%. The K values ranged from 1.44% to 3.16%, with category one as the highest and category three as the lowest. The mean reached 1.44%, and Std obtained 0.66%. The interquartile Q1 reached 1.54%, and Q3 received 2.26%. It can be seen that the concentration of P and K were distributed between classes and were heterogeneous.

### 2.2. LIBS and Vis-NIR Spectra

The LIBS spectra of various organic fertilizer samples are shown in Figure 1b. The different spectra have similar curves and emit the same emission lines. Some atomic emission lines, such as Ca II (393.4 nm, 396.8 nm, 854.2 nm), Ca I (422.7 nm, 616.2 nm), Mg II (280 nm), Mg I (383.2 nm, 518.4 nm), Al II (288.1 nm), and Al I (396.1 nm), were identified through the database of the National Institute of Standards and Technology (NIST: https://physics.nist.gov/PhysRefData/ASD/lines_form.html) accessed on 15 April 2021. Figure 1c shows representative Vis-NIR spectra recorded for various organic fertilizers. In contrast to LIBS, Vis-NIR can identify absorption peaks, and the four varieties also have similar curves. The spectra-wide disparity of the baseline shifts was related to the loading density and the particle size. The absorption peaks associated with the vibration of molecular bonds C–H, O–H, and N–H, which were from water and carbohydrates, were situated between 1350 nm and 1600 nm in the NIR region [27]. The absorption peaks at 1887–2200 nm, which are the most substantial absorbance peaks, can also be seen in the spectra. Overall, the differences between the LIBS and Vis-NIR data spectra for the various categories are difficult to discern. As a result, chemometrics is required for further processing.

### 2.3. Prediction of P and K Using Full Spectra

**Figure 2:**
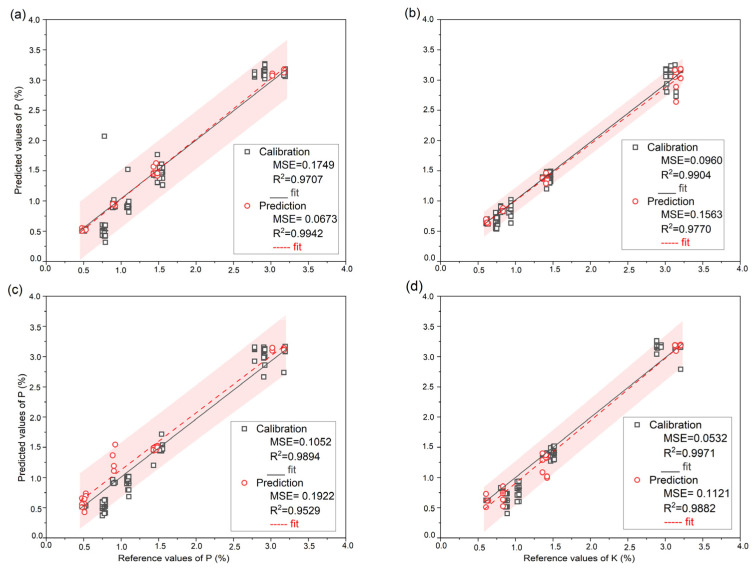
Prediction error plot of PK reference value and predicted value using Extratrees model; (**a**) Prediction error plot of P using full LIBS spectra; (**b**) Prediction error plot of K using full LIBS spectra; (**c**) Prediction error plot of P using full Vis-NIR spectra; (**d**) Prediction error plot of K using full Vis-NIR spectra. The red area means 95% of the prediction interval.

LIBS and Vis-NIR organic fertilizer spectra were correlated with P and K reference data using SVR, PLSR, and Extratrees. The created models utilized 96 samples for calibration and 24 for prediction. We chose to perform k-fold cross-validation on the calibration data with k = 10. Table 1 displays the model statistics for each modeling strategy. Compared to other conventional models, the results obtained using Extratrees were efficient for both calibration and prediction set to predict PK using the complete LIBS and Vis-NIR spectra. Indeed, the detection of P with Extratrees using the LIBS spectra for the calibration set yielded 0.9707 for R^2^c,0.1749% for RMSEc, and the prediction set, R^2^p, was 0.9942, RMSEc produced 0.0673%, and its RPD was 13.15. Meanwhile, Extratrees’ findings for P prediction using Vis-NIR data were R^2^c = 0.9894, RMSEc = 0.1052%, and for the prediction, R^2^p achieved 0.9529, RMSEp = 0.1922% and RPD = 4.60. Figure 2a,c depicts the prediction error of the Extratrees model’s correlation between the P actual values and the P predicted using full LIBS and Vis-NIR. Based on the relationship between the predicted concentrations by Extratrees and their reference concentrations using LIBS and Vis-NIR shown in Figure 2a,c, it can be seen that the calibration curves generated with LIBS outperformed Vis-NIR in terms of prediction.

Regarding K prediction using the LIBS spectra, the Extratrees resulted in 0.9904 for R^2^c, 0.0960% for RMSEc, and its prediction set results were R^2^p = 0.9770, RMSEp = 0.1563%, and RPD = 6.59. When the Vis-NIR spectra were used as input, the calibration result reached 0.9971 for R^2^c, and 0.0532% for RMSEc, while the prediction R^2^p produced 0.9882, RMSEp attained 0.1121%, and RPD reached 9.19. However, compared to P prediction, different phenomena appeared for K prediction as Vis-NIR performed better than data collected by LIBS. Figure 2b,d shows the prediction error plots for the reference value and the prediction value for K using LIBS and Vis-NIR.

### 2.4. Prediction of PK with the Selected Wavelength of LIBS and Vis-NIR, and Their FUSION

Many high-dimensional datasets, such as those obtained with LIBS and Vis-NIR spectroscopy, exhibit a remarkable property known as colinearity, implying that some of the spectral variables are exact linear combinations of others. The existence of colinearity can cause several problems, including overfitting. As a result, it is necessary to compress the correlated or redundant information to preserve the essential data before modeling. Moreover, as seen before, it is crucial to reduce the data before constructing a midlevel fusion approach. This study computed Shap values to interpret and extract the critical features from the Extratrees model since the model predicted PK better using full spectra than any other model (Table 1). The different wavelengths with a powerful positive influence derived from Shap values computation for LIBS spectra (LIBS-Shap) and Vis-NIR ranges (Vis-NIR-Shap) and their fusion (FUSION) were utilized to estimate PK in order to improve quantification findings. To know the minimum number of variables to take among the essential variables with the highest Shap, we calculated the prediction of PK using 5 variables, 10 variables, 20, and 30 variables for Vis-NIR-Shap and LIBS-Shap with Extratrees. The results for PK showed us that 30 wavelengths were the optimal variable and could lead to satisfactory results. The different samples were split into 96 samples for calibration and 24 for prediction. The results of PK prediction using features selected by Shap and the fusion on predicting PK are shown in Table 2.

**Figure 3:**
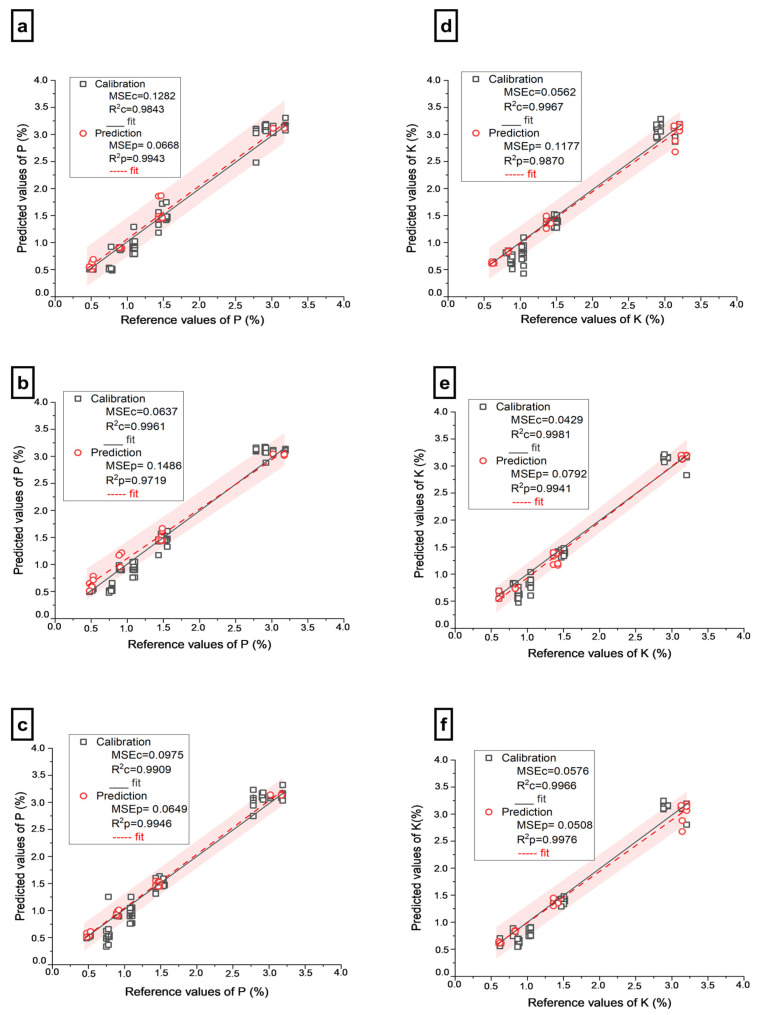
Prediction error plot of PK for calibration and prediction set using Extratrees; (**a**) Prediction error plot of P using LIBS-Shap spectra; (**b**) Prediction error plot of P using full Vis-NIR-Shap spectra; (**c**) Prediction error plot of P using FUSION spectra; (**d**) Prediction error plot of K using LIBS-Shap spectra; (**e**) Prediction error plot of K using Vis-NIR-Shap spectra; (**f**) Prediction error plot of K using FUSION spectra. The red area means 95% of prediction intervals.

A comparison of Table 1 and Table 2 clearly shows an improvement in the models for predicting P content utilizing LIBS-Shap and Vis-NIR-Shap data. The Extratrees model was the most accurate predictor of P for both LIBS-Shap (R^2^p = 0.9943, RMSEp = 0.0668%, and RPD = 13.26) and Vis-NIR-Shap (R^2^p = 0.9715, RMSEp = 0.21486%, and RPD = 5.96) among the models tested. Moreover, when comparing the LIBS-Shap and Vis-NIR-Shap results, it is evident that LIBS-Shap provides superior P prediction results. The prediction error graphs for LIBS-Shap and Vis-NIR-Shap utilized to forecast P are shown in Figure 3a,b. The combination of both sensor data for forecasting P led to better results in comparison to using single-sensor data for all three models, with the highest (Extratrees) reaching 0.9946 for R^2^p, 0.0649 % for RMSEp, and its RPD equaling 13.65. The prediction error curve for FUSION employed to forecast P with Extratrees is shown in Figure 3c. In summary, the ranking of P contents prediction using different spectra was as follows: FUSION > LIBS-Shap > Vis-NIR-Shap.

In contrast to the full data prediction (Table 1), the LIBS-Shap and Vis-NIR-Shap data significantly improved the outcomes of K prediction (Table 2). According to our estimation results, Extratrees produced an excellent prediction result for both LIBS-Shap (R = 0.9870, RMSEp = 0.1177%, and RPD = 8.75) and Vis-NIR-Shap (R^2^p = 0.9941, RMSEp = 0.0792%, and RPD = 13.01). Figure 3d,e shows the prediction error plots for LIBS-Shap and Vis-NIR-Shap used to forecast K. On the other hand, it has also been observed that the fusion of the two datasets produced a better prediction of K (R^2^p = 0.9976, RMSEp = 0.0508, RPD = 20.28) than LIBS-Shap and Vis-NIR-Shap. Figure 3f depicts the prediction error curve for the fusion used to predict K. Overall, the prediction of K contents with different input factors was listed as follows: FUSION > Vis-NIR-Shap > LIBS-Shap.

### 2.5. Discussion

This paper investigated the fusion of Vis-NIR and LIBS spectra on PK prediction in different varieties of organic fertilizers using Extratrees and XAI. The full spectra of LIBS and Vis-NIR were used first to predict PK using SVR, PLSR, and Extratrees models. Table 1 summarizes the calibration and prediction results from the three models. The results for the two sensors demonstrate that Extratrees is the most efficient model. This result was expected since Extratrees’ machine learning model is nonlinear and composed of many decision trees (Ensemble model). The previous study on fertilizer to determine PK was all based on simple linear regression or a simple machine learning model (SVR, PLSR) for both LIBS [12,28,29] and Vis-NIR [15,16]. However, the accuracy and applicability of these simple methods are often less than suitable since they focus only on the lines of interest and discard the spectral information provided by other lines [30].

On the other hand, Extratrees, an ensemble machine learning model, can learn from a large-scale band and decrease variance while increasing bias compared to standard trees [31]. Extratrees have been shown in a few papers to have the potential to lead to favorable results over simple machine learning models [32,33]. In addition to Extratrees, SVR was superior to PLSR in this study for predicting PK using LIBS and Vis-NIR spectra.

**Figure 4:**
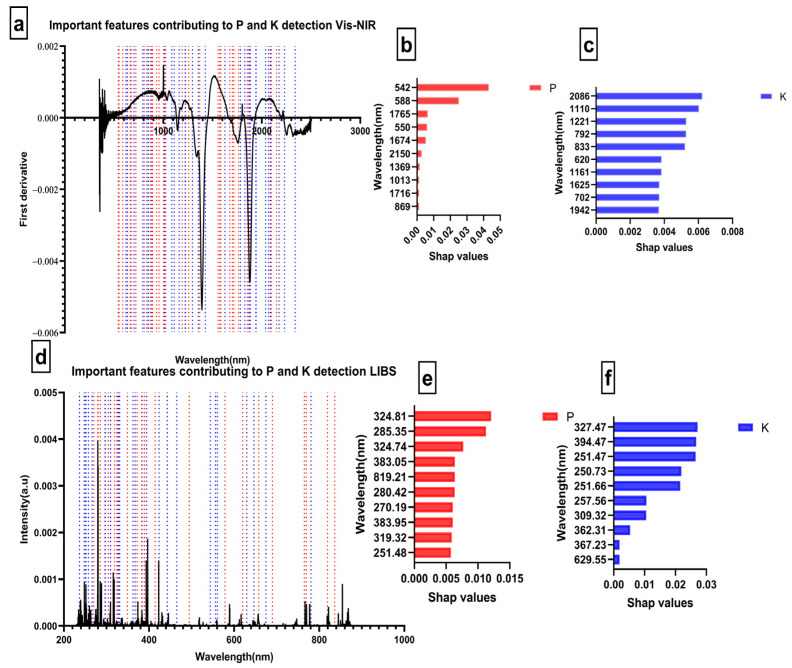
Important features contributing to PK detection using Extratrees; (**a**) Important features for Vis-NIR with a red dot representing P features and a blue dot representing K features; (**b**) Ten features with highest Shap values for P using Vis-NIR; (**c**) Ten features with highest Shap values for K using Vis-NIR; (**d**) Important features for LIBS with the red dot representing P features and a blue dot representing K features; (**e**) Ten features with highest Shap values for P using LIBS; (**f**) Ten features with highest Shap values for K using LIBS.

In addition, to the ability to predict, interpretability is another crucial characteristic of the model. It is pertinent to note that even though the model developed in this study demonstrated excellent nonlinear regression performance. There is still a disagreement as to how to interpret these findings. Thus, Shap was used to obtain directly interpretable data about the PK content predicted by the machine learning model, which avoided the problems caused by “black box” predictions [34]. Model predictions combined with the Shap algorithm produced robust, interpretable, and transparent data, which is critical for chemometrics. As part of this example, we found that the range of variables influencing P prediction in Vis-NIR was between [542–1092 nm]. This region was similar to the iron oxide absorption region presented by Gholizadeh et al. [35]. From this range, we observed that variables at 542 nm and 588 nm with Shap > 0.01 were the most significant features (Figure 4b). While for K detection using Vis-NIR, variables at NIR positions such as 2086 nm, 1110 nm, 1221 nm, 792 nm, and 833 nm were the most impactful, with Shap >0.005 (Figure 4c). A similarity was found between the detected regions and those corresponding to clay minerals and water [9]. The different ranges where the highest contributing features were observed are similar to those reported in [16,36]. However, no information is available in the literature on direct absorption by K and P in the Vis-NIR region.

Regarding the LIBS sensor, the variables significantly contributing to predicting P were 324.81 nm and 285.35 nm with Shap values > 0.01. A number of significant features were also found within the interval [251.48–461.48 nm]. In contrast, 327.47 nm, 394.47 nm, 251.47 nm, 250.73 nm, 251.66 nm, 257.66 nm, and 309.32 nm were substantially linked with the prediction of K.

It should be noted that the wavelengths that drove both P and K’s LIBS prediction were unexpected. Indeed, some literature has reported that P-sensitive wavelengths can be found around 214 nm and 253 nm [12,37]. Furthermore, K was scheduled to be at 766.13 nm or 769.68 [13,14]. However, according to the NIST website and the Kurucz database, the values 324.81 nm and 285.35 nm that contribute to the prediction of P correspond to the elements Fe II [38] and Mg I [39]. This finding leads us to deduce a relationship between P and Mg and between P and Fe. This assertion is endorsed by Fageria [40], who explained that interactions between P and Mg could occur since Mg contributes to activating several enzymes.

Additionally, it has been demonstrated that a small amount of P increases the concentration of Mg [41]. Moreover, two variables among the features, namely 330.9 nm and 324.74 nm, were determined to be P II [42]. Regarding K prediction, the number of reported high contributing variables was mainly in the 230–300 nm wavelength range, which was determined to be KCl and KOH absorption [43].

Full-range spectroscopic data necessitate a complex model development and a long training period. Numerous studies proved that a wide range of features in the Vis-NIR [9] and LIBS [14,44] might make prediction difficult. Thus, we tested the performance of predicting PK using only the variables contributing significantly to the model for LIBS(LIBS-Shap) and Vis-NIR(Vis-NIR-Shap). It turned out that the results for both enhanced the prediction of PK as compared to the full spectrum. Additionally, both sensors’ reduced variables were combined to create a mid-level fusion. It was discovered that combining the two sensors, to some extent, improved the calibration and prediction results. Extratrees’ prediction of P using FUSION data achieved 0. 9946 for R^2^p, 0. 0649% for RMSEp, and 13.65 for RPD, which was marginally better than the predictions using LIBS-Shap and Vis-NIR-Shap, respectively. Similar results were seen for K prediction by Extratrees, with FUSION-Shap (R^2^p = 0. 9976, RMSEp = 0.0508%, RPD = 20.28) outperforming Vis-NIR-Shap and LIBS-Shap in terms of precision. The results obtained from combining Vis-NIR and LIBS were expected because data fusion approaches have demonstrated improved outcomes in predicting some other materials [21,45].

Overall, the experimental findings demonstrate that the computation of Shap values may aid in a better understanding of the model and the identification of high-impact wavelengths that can enhance the detection of PK in organic fertilizers utilizing LIBS and Vis-NIR technologies. A further finding of the study was that the combination of both sensors might be used to provide a more accurate and reliable prediction of PK, although additional samples may be required to support this idea. XAI has the potential to significantly impact chemometrics by promoting confidence in analyses, especially in applications where decisions made based on the analysis have significant consequences for individuals or society. Moreover, as XAI can help to merge LIBS and Vis-NIR data, it can therefore enable the creation of a powerful new sensor technology that could be helpful in characterizing elements in complex samples. Lastly, the study will contribute to environmental conservation and the viability of local agriculture.

## 3. Material and Methods

### 3.1. Samples

The College of Biosystem Engineering and Food Science at Zhejiang University in Hangzhou (China) provided the organic fertilizers used in this study. Four distinct categories of organic fertilizers were utilized in this investigation. For category one (1), the raw materials are fermented and digested by earthworms. At the same time, category two (2) is a mix of rice straw and earthworm manure. Category three (3) raw materials are pure sheep manure fermented and decomposed by microbial fungus. The last category (4) is made through the fermentation and decomposition of chicken manure as raw materials. Each type was composed of thirty samples, resulting in a total of 120. The various fertilizer samples were dried and ground into powders in a grinder, then sieved through a 60-mesh screen. Finally, pellets with a 30 mm diameter and a 2 mm thickness were created with a pressure machine (pressure = 20 MPa, time = 15 s) for all 120 samples.

### 3.2. Chemical Reference Values Measurement through ICP-MS

The exact concentrations of phosphorus(P) and potassium(K) were determined using inductively coupled plasma mass spectrometry (ICP-MS), which is an effective and widely used technique for analyzing environmental materials for multiple elements and isotopes. Each organic fertilizer was digested in 2.5 mL of nitric acid (HNO_3_), 1 mL of hydrogen peroxide(H_2_O_2_), and hydrofluoric acid (HF). We then combined these solutions, microwaved (MARS 6, CEM, Mathews, VA, USA) at 240 °C, 80 bar for 10 min, then heated and cooled. Approximately 0.25 g of each sample was weighed and placed into 50 mL Falcon tubes along with 25 mL Milli-Q water. All of the samples were centrifuged, and the supernatants were transferred to Falcon tubes after being agitated for 1 h on an orbital shaker. If particles could be detected in the supernatant, they were filtered using filter papers. The extractions were performed in triplicate to guarantee total analyte extraction, and the Chinese national standards (CNS), which are technical norms issued by the National Standardization Administration of China, were employed as reference materials. In the end, the solution was analyzed by ICP-MS (ELAN DRC-e, PerkinElmer, Beijing, China) to measure the amount of P and K, especially isotopes P-31 and K-39. It should be noted that the dynamic reaction cell (DRC) of the ICP-MS is filled with argon gas in order to remove interferences. The obtained result of P is reported as the percentage (%) of pentoxide oxide (P_2_O_5_), commonly referred to as the element phosphate, in order to distinguish it from phosphorus (P). In contrast, K is reported as a percentage of potassium oxide (K_2_O), called potash. However, we will use P and K in the whole manuscript, referring to P_2_O_5_ and K_2_O.

### 3.3. Spectral Measurement and Preprocessing

#### 3.3.1. LIBS Measurement and Preprocessing

**Figure 5:**
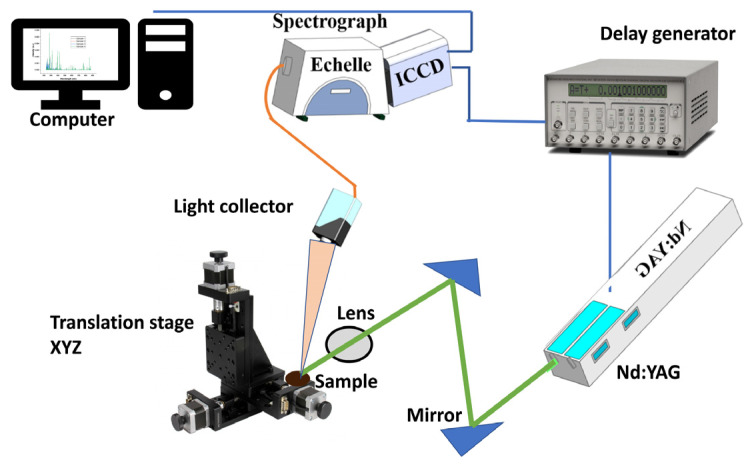
LIBS equipment setup.

In this study, the LIBS equipment (Figure 5) employed to generate plasma was made of a Q-switch pulsed laser (Vlite 200, Beamtech, Beijing, China) with parameters such as maximum energy, pulse duration, and wavelength, the same as reported by Zhao et al. [7]. The obtention and the record of the emission spectra have been made possible through a spectrograph (ME5000, Andor, Belfast, UK) and an image charge-coupled device (ICCD- DH334T-18U-03, Andor, Belfast, UK). At the same time, the delay generator helped to control the ICCD camera and the laser Q-switch. The overall system has a spectral range of 229–876 nm. The LIBS spectra were obtained by applying 16 laser pulses to five randomly chosen positions of each sample. A total of 80 spectra were obtained and averaged for each sample to eliminate heterogeneity. The median filter was used to minimize noise first. Then, area normalization was employed to normalize the smoothed spectra to the same scale. In the end, iteratively reweighted penalized least squares were used to remove the spectral background.

#### 3.3.2. Vis-NIR Measurement and Preprocessing

Vis-NIR spectra were collected by suspending a 50 W halogen lamp of a Fieldspec3 spectral analytical device (Analytical Spectral Devices, Boulder, CO, USA) directly above each experimental Petri dish. However, Unlike LIBS, which uses pelletized samples, Vis-NIR uses raw and unpelletized samples. The spectrometer was calibrated once per fifteen measurements with a white plate using a Spectralon (Malvern Panalytical Ltd., Malvern, UK), and the sample was measured three times. In the end, 360 spectra were acquired and averaged to obtain 120 spectra. The multiplicative scatters correction (MSC) and the first-order derivative were applied to eliminate the baseline offset [9].

### 3.4. Chemometrics

#### 3.4.1. Conventional Machine Learning

Two conventional machine learning models were used to estimate the PK content: SVR and PLSR. Many papers have discussed and explained these two algorithms [9,46]. The best parameters for each model were found through grid search computation.

#### 3.4.2. Ensemble Machine Learning

Extremely Randomized Trees, also known as Extratrees, is a machine learning ensemble algorithm proposed by Geurts et al. [31]. Extra trees generate a massive number of decision trees from the training dataset and use majority voting for classification. In contrast, regression uses the average of the different predictions for each decision tree. Contrary to Random Forest, which uses the tree bagging step to obtain the training subset for each tree, Extra tree uses the whole training set. Furthermore, it chooses the best feature and value to split the node, making the extra trees less likely to overfit and reporting better results. The Extratrees model was used to detect the PK contents.

### 3.5. Wavelength Selection through Shapley Additive Explanation Values

XAI helps to visualize, explain, and interpret the machine learning models. There are many XAI approaches [47]. However, in this study, we will focus on Shapley additive explanation values (Shap), which Lundberg and Lee [48] described. The Shap algorithm is a combination of game theory [49] and local explanation [50]; its primary purpose is to know the contribution of each feature in a particular prediction. In order to better allocate the contribution of each feature $\left(\phi_{i}\right)$, its Shapley values were calculated through:

(1) $$\phi_{i}=\underset{S\subseteq F\{i\}S}{\sum }\frac{\left|S\right|!\left(F-\left|S\right|-1\right)!}{F!}\left[f_{SU\left\{i\right\}}\left(x_{SU\left\{i\right\}}\right)-fs\left(xs\right)\right]$$

The purpose is to retrain all features subsets S of the model. S is included in variable F, which represents all features, $f_{SU\left\{i\right\}}$ is the model used to train the feature subset, and fs is the model used to train the retains feature. To compare both predictions $f_{SU\left\{i\right\}}\left(x_{SU\left\{i\right\}}\right)-fs\left(xs\right)$ were calculated, and this is repeated for all subsets. Furthermore, the explainability of the additive feature attribution represented by a linear function g was determined through:

(2) $$g\left(z^{\prime }\right)=\phi_{0}+\overset{M}{\underset{i=1}{\sum }}\phi_{i}z_{i}^{\prime }$$

The coalition of the vector $Z^{\prime }$ is between the interval {−1,1}, and M is the number of features. Moreover, as the Shap values only emphasize the importance and mention whether the importance has a positive or negative impact on the model, we modified it in order to be able to extract relevant features. In order to be able to extract features, we will calculate the correlation between Shap $\phi_{i}$ and the data used to calculate the Shap noted Xi. Both must have the same shape size (P*n) and should have the same feature number M. Then, for each feature number Mi, we calculate the correlation noted in Corr ($\phi_{i},Xi$). In the end, we will pose a constraint to select only Corr > 0 in order to select the relevant features.

### 3.6. Data Fusion Approach

Data fusion aims to create a more comprehensive and detailed set of information by integrating data from multiple sources. There are three methods of implementing data fusion, namely low-level, mid-level, and high-level, according to the fusion level between the different data forms. In this study, we will focus on mid-level fusion, which is achieved by merging the features extracted from the Vis-NIR and LIBS spectra. In order to obtain the best features for LIBS and Vis-NIR, the Shap values were computed to extract the essential features that contribute the most to predicting P and K in organic fertilizer. The variables with the highest Shap value were considered essential.

**Figure 6:**
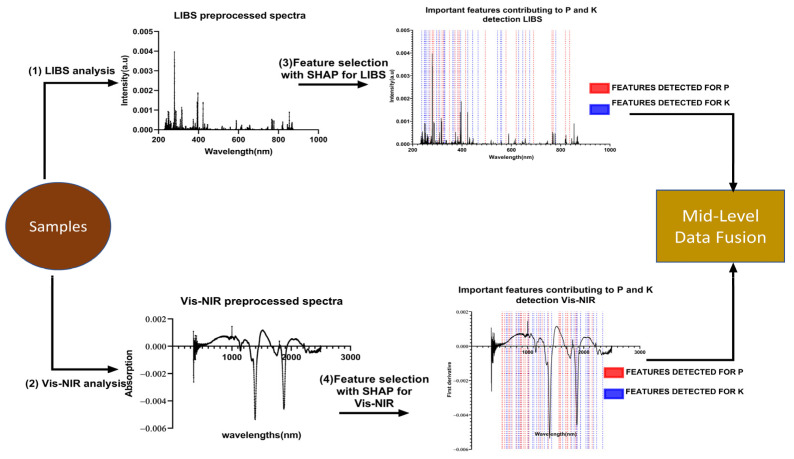
Flowchart midlevel fusion.

Overall, the features with the highest Shap values for LIBS, called LIBS-Shap, were concatenated to the features with the highest Shap values for Vis-NIR, called Vis-NIR-Shap, to improve the quantification of P and K. The Scheme of the data fusion process of mid-level using Shap values can be found in Figure 6.

## 4. Conclusions

This study aimed to explore the potential of explainable AI on chemical analytics in predicting PK in various organic fertilizers using LIBS and Vis-NIR sensors. First, the full spectra of LIBS and Vis-NIR were used to predict PK using Extratrees, SVR, and PLSR. The quantification results showed that Extratrees provided better results than SVR and PLS for both sensors. Secondly, XAI through Shap computation was used to explain Extratrees and select important characteristics. The results of different features demonstrated that P’s important features were mainly at the visible part while K’s main characteristics were located at the NIR part for Vis-NIR. For LIBS, the spectra’s main features for both P and K were between [251.48–461.48 nm]. The computation of Shap values assisted in interpreting the model and helped select features, improving the detection performance of PK for both sensors (LIBS-Shap and Vis-NIR-Shap). Meanwhile, the fusion of the reduced spectra obtained with the two sensors (FUSION) resulted in slightly better results than a single sensor. The performance of the model for P with FUSION achieved 0.9946 for R^2^p, 0.0649% for RMSEp, and 13.65 for RPD, slightly better than LIBS-Shap (R^2^p = 0.9943, RMSEC = 0.0668% and RPD = 13.26) and Vis-NIR-Shap data (R^2^p = 0.9719, RMSEp = 0.1486%, and RPD = 5.96). While for K prediction FUSION with R^2^p = 0.9976, RMSEp = 0.0508% and RPD = 20.28 outperformed better than Vis-NIR-Shap (R^2^p = 0.9941, RMSEp = 0.0792%, and RPD = 13.01) and LIBS-Shap (R^2^p = 0.9870, RMSEp = 0.1177%, and RPD = 8.75). Finally, the outcomes of these results serve as a step toward understanding the usefulness of explainable AI in analytical chemistry to build a robust model for detecting chemical elements using LIBS, Vis-NIR, and fusion spectra.

## Figures and Tables

**Table 1:** Prediction of PK with full spectra LIBS and Vis-NIR using PLSR, SVR, Extratrees.

			Calibration		Prediction		
Data	Elements	Models	R^2^c	RMSEc	R^2^p	RMSEp	RPD
LIBS	P	SVR	0.9774	0.1535	0.9736	0.1439	6.15
	P	PLSR	0.9547	0.2174	0.8787	0.3086	2.87
	P	EXTRA	0.9707	0.1749	0.9942	0.0673	13.15
	K	SVR	0.9618	0.1918	0.8430	0.4086	2.52
	K	PLSR	0.7406	0.4998	0.8379	0.4152	2.48
	K	EXTRA	0.9904	0.0960	0.9770	0.1563	6.59
Vis-NIR	P	SVR	0.9849	0.1255	0.9466	0.2047	4.32
	P	PLSR	0.9806	0.1424	0.9008	0.2790	3.17
	P	EXTRA	0.9894	0.1052	0.9529	0.1922	4.60
	K	SVR	0.9866	0.1135	0.9722	0.1719	5.99
	K	PLSR	0.9862	0.1152	0.8894	0.3429	3.00
	K	EXTRA	0.9971	0.0532	0.9882	0.1121	9.19

R^2^c: Coefficient of determination for calibration; R^2^p: Coefficient of determination for prediction; RMSEc: Root mean square error for calibration; RMSEp: Root mean square error for prediction; RPD: Residual prediction deviation.

**Table 2:** Prediction of PK with PLSR, SVR, and Extratrees using features selected by Shap computation for LIBS and Vis-NIR and the fusion spectra.

			Calibration		Prediction		
Data	Elements	Models	R^2^c	RMSEc	R^2^p	RMSEp	RPD
LIBS-Shap	P	SVR	0.9806	0.1423	0.9732	0.1450	6.11
	P	PLSR	0.9369	0.2566	0.8812	0.3054	2.90
	P	EXTRA	0.9843	0.1282	0.9943	0.0668	13.26
	K	SVR	0.9798	0.1396	0.9500	0.2306	4.47
	K	PLSR	0.8729	0.3498	0.7987	0.4626	2.22
	K	EXTRA	0.9967	0.0562	0.9870	0.1177	8.75
Vis-NIR-Shap	P	SVR	0.9881	0.1114	0.9605	0.1762	5.02
	P	PLSR	0.9564	0.2134	0.8531	0.3396	2.60
	P	EXTRA	0.9961	0.0637	0.9719	0.1486	5.96
	K	SVR	0.9917	0.0893	0.9838	0.1311	7.86
	K	PLSR	0.9832	0.1273	0.8871	0.3465	2.97
	K	EXTRA	0.9981	0.0429	0.9941	0.0792	13.01
FUSION	P	SVR	0.9843	0.1280	0.9784	0.1416	6.48
	P	PLSR	0.9664	0.1873	0.9046	0.2737	3.23
	P	EXTRA	0.9909	0.0975	0.9946	0.0649	13.65
	K	SVR	0.9915	0.0903	0.9872	0.1168	8.82
	K	PLSR	0.9746	0.1564	0.8838	0.3515	2.93
	K	EXTRA	0.9966	0.0576	0.9976	0.0508	20.28

R^2^c: Coefficient of determination for calibration; R^2^p: Coefficient of determination for prediction; RMSEc: Root mean square error for calibration; RMSEp: Root mean square error for prediction; RPD: Residual prediction deviation.

## Data Availability

The data presented in this study are available on request from the corresponding author. The data are not publicly available due to privacy.
